# ‘Worth … the Nightmares’? A Qualitative Study of Early‐Career Healthcare Workers' Wellbeing and Career Decisions After Starting Work During Covid‐19

**DOI:** 10.1111/tct.70120

**Published:** 2025-06-19

**Authors:** Megan E. L. Brown, Bryan Burford, Christopher Price, Yu Fu, Gillian Vance

**Affiliations:** ^1^ School of Medicine Newcastle University Newcastle‐Upon‐Tyne UK; ^2^ Population Health Sciences Institute Newcastle University Newcastle‐Upon‐Tyne UK; ^3^ University of Liverpool Liverpool UK

**Keywords:** Covid‐19, medical education, NHS, nurse, paramedic, wellbeing, workforce

## Abstract

**Background:**

The Covid‐19 pandemic placed unprecedented pressure on healthcare systems, education and individual professionals—notably, newly qualified front line nurses and paramedics. While we know wellbeing was negatively affected, qualitative exploration of workplace experiences and how these impact wellbeing and shape career decision making is lacking. This is a critical gap in the literature, given the current healthcare workforce crisis and a need for evidence‐based educational reform to better support learners in the workplace. This study explores the experiences of nurses and paramedics during the pandemic and how these influenced wellbeing and career decision‐making.

**Methods:**

We adopted an interpretivist, qualitative approach, and conducted semi‐structured interviews with seven newly qualified nurses and two senior paramedics. We utilised reflexive thematic analysis to explore and analyse data, considering the impact of starting work during Covid‐19.

**Findings:**

Our findings demonstrated the significant impact of the pandemic on wellbeing and career decision‐making. Key issues included a turbulent transition into practice, shaped by increased clinical pressures (including high patient numbers, and workforce shortages) and reduced support at organisational and interpersonal levels. Participants described a shift in career values, with greater emphasis on wellbeing as a determinant for job decisions. Many reported re‐evaluating their careers, prioritising roles or teams that offered better support and manageable workloads, or considering leaving healthcare altogether.

**Conclusion:**

This study suggests a need for educational policy and practice to consider how workplace experience impacts wellbeing and career decision‐making. Beyond indicating the need for further research, this study acts as a critical conversation regarding future workforce planning.

## 1 Introduction

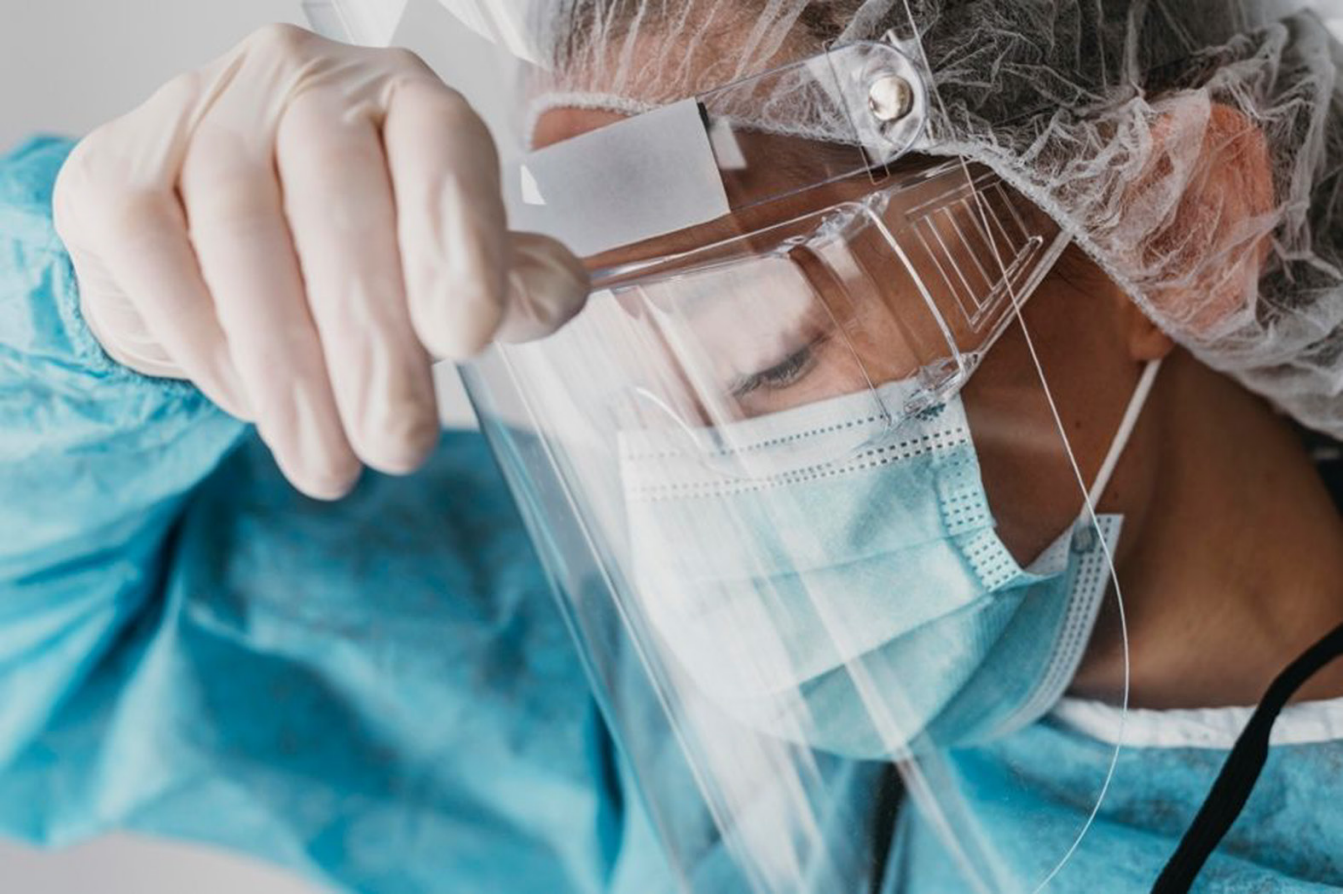

The Covid‐19 pandemic had a profound impact on healthcare professionals' wellbeing, with implications for workforce sustainability, given wellbeing is connected to retention, performance, and organisational functioning [1, 2, 3]. Nurses and paramedics, as front‐line workers, were among the most affected [4, 5, 6].

Despite evident strain, little is known about how the lived experiences of early‐career nurses and paramedics during Covid‐19 relate to workforce sustainability. Quantitative studies have highlighted pandemic factors influencing ‘turnover intention’ e.g., fear, stress, and lack of support [7]. International qualitative studies exploring the experiences of new practitioners during Covid‐19 have highlighted that new graduates felt ill‐prepared for practice [8], experienced fear and isolation [9], how new nurses learned to look for hope [10] and have explored undergraduate nursing [11] and paramedicine [12] students' experiences of learning and career plans.

Literature also identifies a widespread lack of support for early‐career professionals [13, 14, 15, 16]. Some research has begun to explore factors influencing retention post‐Covid, within nursing particularly, e.g., job security [17] and supportive teams [18], but, to the best of our knowledge, no studies have considered how wellbeing during the transition to practice amidst the pandemic is affecting longer‐term career decisions within nursing and paramedicine. Understanding how this context has shaped nurses and paramedics is important for informing retention strategies [18].

This study asked: *How has the experience of starting work during the Covid‐19 pandemic influenced the wellbeing and career decisions of nurses and paramedics?* Our intention was not to compare respective experiences, but to explore transferable insights regarding the long‐term impact of pandemic‐era transition into practice. We focused on new nurses and paramedics as they are at a pivotal career point, where decisions carry weight for both their futures, and the system's. This builds on our previous research into doctors' experiences of starting work during the pandemic [19, 20].

## 2 Methods

### 2.1 Research Approach

We adopted an interpretative qualitative approach to explore the subjective experiences, thoughts and feelings of our target populations regarding wellbeing and career decision‐making.

### 2.2 Data Collection

We wished to recruit a convenience sample of nurses and paramedics who started work during the pandemic. Participants were nurses in four NHS Trusts and paramedics in one Ambulance Trust in one region of England. Trusts were located in the North East of England and included a mix of urban/rural service areas. Participants worked across a range of acute and community settings. The study focused on early‐career experiences within these varied contexts. Participants were recruited through emails sent by Trusts and adverts for the study posted to relevant newsletter/staff social media groups.

Recruitment proved challenging due to service pressures. Despite these difficulties, we successfully recruited seven nurses who either qualified during the pandemic or were in the final months of training as Covid‐19 began. All were in clinical roles during this period. Unfortunately, no paramedics initially came forward. We expanded recruitment to include senior paramedics who had experience supporting new paramedics during Covid‐19. This enabled us to recruit two paramedics.

We used principles of information power [21] to guide decisions regarding sample sufficiency. As our study had a narrow focus, high‐quality dialogue, and a clear analytic approach, we judged nine participants sufficient to address our aim. Identifying recurrent patterns during coding supported this.

All nine participants participated in an online interview (mean duration 40 min) with MB/SM (postdoctoral researchers). Interviews focused on participants' experiences of starting work during Covid‐19, wellbeing, career to‐date, aspirations and plans, with guides developed from the team's knowledge of transition to practice literature. For senior paramedics, questions were adapted to encourage them to reflect on experiences of supporting new paramedics during Covid‐19 and perceptions of junior colleagues' wellbeing and career plans (e.g., questions rephrased in third person, and as reflection on observations rather than inquiring about direct experiences). While these are not first‐person accounts, we believe senior perspectives still offer valuable insights into organisational and interpersonal conditions affecting newly qualified colleagues.

Interview questions used for the nurses who began work during the pandemic are available (Appendix A).

### 2.3 Data Analysis

We used Braun and Clarke's [22] reflexive thematic analysis. MB transcribed and read all transcripts for immersion, then created descriptive codes in Atlas.ti, using participants' words where possible. She organised codes into draft themes, reviewed them alongside linked quotations, and developed names and definitions. The overall narrative was refined through team discussion. Throughout the analysis, an audit trail documented decisions and theme development. Memos documented reflections, and regular team meetings supported interpretative rigour.

### 2.4 Reflexivity

Reflexivity is essential to Braun and Clarke's approach [23]. Authors reflected on their positionality and relationship to the topic and data (Table 1).

**TABLE 1:** Team reflexivity statement.

GV and CP are clinical academics, who both worked clinically as doctors during the Covid‐19 pandemic. BB's background is in psychology, while MB is clinically trained but non‐practicing. Several members of team have been involved in research focussed on the wellbeing of healthcare professionals (GV, BB, MB) and workforce priorities (GV, BB, MB). Though our professional associations and backgrounds offer insight into the realities of working during the pandemic, and theoretical knowledge in relation to wellbeing and workforce, it is important to note that our clinical experiences are within medicine, rather than in nursing or paramedic services. However, CP has led research evaluating the effectiveness of ambulance training and paramedic views about new care pathways, and BB has led research exploring the concept of professionalism with paramedics. To further account for this, we have actively sought out literature from these fields to situate our analysis in what is more broadly known within the field.

## 3 Results

We identified three themes from our thematic analysis:

1. Heighted challenge of entry into practice
2. Trauma and lack of support
3. Wellbeing as a key career decision‐making value

Within each theme, we also identified several subthemes, which we introduce and explore throughout this section. We have labelled quotes with a participant number (1–9) and their role (nurse, or paramedic).

### 3.1 Heightened Challenge of Entry Into Practice

The transition from student to healthcare professional is known to be challenging. Several of our participants were in their final months of training as the pandemic began, while others had just qualified. For all, the pandemic fractured the usual boundary between student and practitioner, creating an uncertain and pressured entry into clinical work.

For the people who qualified during the pandemic … that transition period … I can't think of any way in which it would be easier … it was more difficult because of [Covid]. Participant 6, Paramedic

Pandemic‐related changes to care, though necessary, unintentionally hindered participants' ability to work effectively. They described uncertainty in clinical decision‐making when faced with unfamiliar, complex cases they felt unprepared for.

The whole day was just muddling through and trying to make what you thought was the right decision … doing that based on six months experience or less … an impossible thing to do.

Participant 7, Paramedic

I felt thrown out there … people were there … . with complications I knew nothing about. Participant 8, Nurse

#### 3.1.1 Lack of Learning Spaces

Participant 3 raised concerns about unsafe practice during the pandemic, citing the abrupt shift from simulated learning to real clinical work without prior practice. They also noted limited support for reflection on new experiences due to an overwhelming workload.

You haven't got that safe space of practicing your clinical skills. You're doing it for the first time in a real life situation … It was quite scary at times Participant 3, Nurse

Similarly, Participant 2 noted that educational support during home visits, normally provided by seniors, were absent.

It made it [home visiting] a lot more stressful and I don't really think I got as much out of it as what I probably would have done had it not been Covid … I feel like I missed out on some support … I don't think there was enough time either. Participant 2, Nurse

### 3.2 Trauma and Lack of Support

Our second theme focused on how emotional stress, lack of organisational support, and mental health stigma combined to affect wellbeing. We heard many stories of trauma and extreme distress. The uncertainty associated with Covid‐19, both its risks and impact on work, created fear during the early pandemic. This included fear of others' mortality, of one's own mortality, and from a changing work context.

That was when we thought all the people who were working with the Covid patients were going to die. Participant 1, Nurse

It was really anxiety provoking. You weren't able to switch off … you were constantly worrying about [redeployment] with no experience and also it wasn't clear what was expected of you when you were moving over. Participant 4, Nurse

#### 3.2.1 Moral Injury and Vicarious Harm

Participant 1 takes their comment further, highlighting the moral injury they experienced as a result of ‘really traumatic deaths’, especially in intensive care, and how what they perceive as poor management responses exacerbated issues.

To cool down patients in extreme heat, for example, staff resorted to makeshift solutions.

It reached 36 degrees and we were in full gowns … we were filling gloves with water and then freezing them because we had no other way to cool patients … we got given a box of ice lollies, that was the management's nod towards helping … we couldn't eat them because we were in PPE all day, so we used to stuff them in patients pillows … if you forgot and they popped, you'd find this pink sludge running out of your patients pillow. Participant 1, Nurse

#### 3.2.2 Organisational Failures

Almost universally, participants felt they did not receive adequate support from organisations. Comments relating to broader organisational failures often focused on the lack of adequate Personal Protective Equipment (PPE).

I think there's a lot of failings with PPE, and we didn't have sufficient PPE on Covid HDU … we were meant to have hair nets. We had shoe protectors instead. Participant 3, Nurse

Comments regarding a lack of support for newly qualified staff spanned nursing and paramedic practice. Comments concerned a lack of clinical, and interpersonal, support at institutional and managerial levels.

The focus was very much on surviving the day … they weren't focused on … what was actually happening on a day‐to‐day basis in terms of practice and support. Participant 7, Paramedic

I can't say that I had a manager who seemed to care apart from maybe in ITU, who are used to people being traumatized a little bit more. Participant 1, Nurse

### 3.3 Long‐Term Impact on Wellbeing

There were many comments about wide‐scale burnout and poor morale.

Morale is bad. I think morale is bad in the ambulance service and has been for a little while now and I think that will impact on their [Newly Qualified Paramedic's] well‐being. Participant 6, Paramedic

Understandably, some of our participants also experienced general anxiety outside of their work settings that required treatment in the aftermath of the pandemic.

I used to get up in the night because I thought I'd fallen asleep on night shift… I'd wake up convinced there were patients in the next room … so I get up, get dressed … into the next room in a panic … I did that for over a year. Participant 1, Nurse

Compounding and worsening negative impact was the stigma associated with mental illness within clinical environments. Participants below describe avoiding disclosure.

If I called in sick, I would say I would have a physical symptom but I would know it was mental health… if I said … it's been traumatic and I'm struggling, I wouldn't have any sympathy. Participant 1, Nurse

I was just so worried they'd [the clinical team] would think less of me … I hid it and hid it and hid it until it all came rushing out. Participant 9, Nurse

### 3.4 Wellbeing as a key Career Decision‐Making Value

This final theme illustrates how Covid‐19 influenced newly qualified healthcare staffs' career decisions. Career decisions are, and remain, complex and multifaceted. While many factors were considered, our data suggest that wellbeing has become a more consciously prioritised value in decision‐making, particularly in light of participants' experiences during the pandemic.

#### 3.4.1 Considering a Future in Healthcare

Several participants described moments when they considered leaving clinical work. Intense pressures and disrupted support systems led new practitioners to doubt the safety of independent practice.

I kept thinking, is this really what I wanna' do? Because if I'm feeling like this as a student, what's it gonna' be like when I'm qualified?… as a student you have that safety net around you, perhaps more so than you would do as a registered nurse … for those last three months of my training, I really had serious doubts as to whether I wanted to carry on with it or not. Participant 3, Nurse

Participant 1 described wishing to either advance to a more senior Band 6 role or to leave nursing. In the NHS, different ‘Agenda for Change’ [24] pay bands reflect levels of responsibility and seniority. Band 6 refers to a more senior role, offering higher pay compared to lower bands. Here, Participant 1 is describing, therefore, issues with putting up with poor conditions for poor pay.

My resolution this year was to either get a Band 6 role, or to leave nursing, one of the two. I wasn't sure which way it was going to go … I was looking at whether I should speak to a careers adviser about other roles… private care, or retraining. Participant 1, Nurse

Ultimately, all participants remained in clinical roles and many valued skills and experiences from the pandemic. Participant 9 developed a new appreciation for research, while Participant 1 shifted from day surgery to caring for acutely unwell patients.

It really tested me, but I don't think I'd be as interested in [pursuing] research if I hadn't seen how reliant we were on it … during those lockdown briefings. Participant 9, Nurse

It's definitely completely changed my career path. I've gone from day surgery where the patients aren't meant to be sick … to sick patients in an ITU. Participant 1, Nurse

#### 3.4.2 Changing Roles

However, the experience had, for many, changed the shape or nature of their role. Some were now working in research, some managerially and most had changed institutions and posts.

… because of Covid … I was looking at where to go, I did look at general paediatric wards, I looked at theatre and then saw this research job… Participant 2, Nurse

Even though I wanted to leave, I know that … working for the NHS is where I'm gonna' be until I retire that … you just sort of hope that you'd have had a slightly better start … I am quite determined that I wanna' probably move on in a year or so's time and maybe start to think about other opportunities. Participant 3, Nurse

Though not universally noted in our data, Participant 7 made an interesting observation that the impact of Covid‐19 on the career decisions of newly qualified staff might be traced back to a transition to practice which did not follow ‘standard’ processes.

I think we've missed out on a smooth transition from students to NQP … it's just been frustrated with a really, really volatile experience, I think … for me personally, at that point in my career, that wouldn't have been a positive thing for me, and I can completely see … why somebody would need to reconsider that career. Participant 7, Paramedic

#### 3.4.3 Emphasis on Wellbeing in Career Decision‐Making

Prominent in participants' decision‐making was the value now placed on wellbeing. The pandemic prompted many early‐career participants to reflect on what mattered most in their careers.

One thing it made me realise is that you can't put your life on hold … we had young people dying … it was really devastating, but it made me think about what is important … I reached a snapping point where I made this decision that I could only give nursing so much, it couldn't be all of me. Participant 8, Nurse

Personal wellbeing was given particular weight as a guiding value in choosing a new post or role.

It [Covid] made newly qualified paramedics think a lot harder about leaving, other alternative career options … that overlaps with wellbeing … shift[s] … emergency services takes a toll … people are a lot more aware of that Participant 6, Paramedic

Nurses don't feel like it's worth it anymore, especially after everything we've been through … when you could do something else which pays just as well and doesn't give you nightmares. Participant 1, Nurse

## 4 Discussion

We explored how starting work during the Covid‐19 pandemic influenced the wellbeing and career decisions of nurses and paramedics. In interviews with seven early‐career nurses and two senior paramedics reflecting on newer colleagues experiences', we heard how pandemic‐era pressures were intensely felt by those new to clinical work. Participants described trauma, distress, and a lasting impact on wellbeing. Three years on, many are re‐evaluating what they value in their careers, placing greater weight on wellbeing. Below, we consider how our findings add to existing literature and identify implications for educators and healthcare systems.

*Participants described trauma, distress, and a lasting impact on wellbeing*.

Our findings underscore the fragility of the transition from education to practice, which was made more difficult by pandemic conditions. Clark and Springer [25] highlighted how the transition from learner to nurse is a formative time for developing commitment to the profession, which can be supported by developing relationships with other staff and actions which imbue a sense of value. For our participants, interpersonal support was disrupted by the pandemic, and many interpreted a lack of organisational support (e.g., lack of PPE) as disregard for their safety, eroding trust.

Burford et al.'s [19] found that informal interpersonal support was vital for new doctors during the pandemic, but constrained by social distancing. Similarly, Murray, Sundin, and Cope [26] link disrupted socialisation and mentorship to poor retention in nursing. Our study reinforces these findings, and suggests that wellbeing is a key mechanism through which lack of support affects retention. Clinical educators can support new staff by advocating for structured peer communities [27], emotional safe spaces [28], and regular team contact [29], even when broader systemic change may be slow.

While prior literature on ‘turnover intention’ (e.g., [7]) highlights the impact of stress and poor support, less is known about how these experiences shift workforce values. Our study suggests that wellbeing has become a central concern in early‐career decision‐making. This aligns with nursing literature connecting job satisfaction and attrition [30, 31] and with UK workforce trends favouring flexible or hybrid work for improved work‐life balance [32].

Although some studies predict increased attrition, our findings suggest a more complex picture. Rather than exiting healthcare, many participants are reshaping their roles around wellbeing. Reframing retention through the lens of role modification—not just turnover—may offer a more accurate view of long‐term workforce change post‐pandemic. While many professionals still intend to stay within the NHS, their ability to do so depends on adapting roles to manage ongoing pressures and the lasting emotional impact of high‐stress events like Covid‐19. Though the acute phase has passed, the strain it caused has exacerbated pre‐existing issues such as burnout [5], with stressors like staff shortages likely to persist [33]. Without adequate support, some professionals may still consider leaving. Employers, policymakers, and educational bodies should recognise this shift in values, particularly among newly qualified staff, and work to improve flexibility, wellbeing support, and meaningful career development opportunities.

*Rather than exiting healthcare, many participants are reshaping their roles around wellbeing*.

Further, careers guidance in clinical education generally lacks personalisation in the context of wider preferences for working life—exploring career coaching as a tailored, individualised approach and offering advice on flexible career paths and opportunities, is likely to support career decision‐making for students [34]. Educators could consider revising, or advocating for changes to, traditional measures of success which focus on productivity and clinical skills. A more holistic approach, where wellbeing, emotional health, and job satisfaction are also included, may better align with the changing values of new professionals.

### 4.1 Limitations

We struggled to recruit, and so our sample size is relatively small, though adequate for our research aims. We used convenience sampling, which may limit diversity of perspectives. In addition, our sample included a mix of participants at slightly different stages in early careers. For example, while all nurses had begun or were completing clinical work during the pandemic, their exact points of transition varied, and paramedic data reflect the perspectives of senior staff. This affects transferability and our ability to compare across groups.

Despite this, we believe there is value in sharing these findings—we have spent time ensuring depth of expression and believe these findings could act as a critical conversation‐starter. We hope this study will inspire further research on the intersection of wellbeing, turnover, and postgraduate education for nurses and paramedics.

### 4.2 Conclusion

This study explored how starting work during the pandemic affected the wellbeing and career decision‐making of nurses and paramedics. Participants described entering practice during a period of high pressure and low support, with lasting effects on wellbeing and professional priorities. Wellbeing has become central to how these professionals navigate their careers, highlighting the need for clinical educators and organisations to prioritise it as a foundation of workforce sustainability. Educational settings, both undergraduate and postgraduate, should actively support wellbeing and flexible career planning.

While limited by a small sample, these findings are a starting point for future longitudinal research into the lasting impact of the pandemic on healthcare careers.

*These findings are a starting point for future longitudinal research into the lasting impact of the pandemic on healthcare careers*.

## Author Contributions

**Megan E. L. Brown:** project administration, formal analysis, methodology, conceptualization, investigation, writing – original draft, writing – review and editing, software. **Bryan Burford:** conceptualization, methodology, formal analysis, supervision, funding acquisition, writing – review and editing, resources, project administration. **Christopher Price:** conceptualization, supervision, formal analysis, project administration, funding acquisition, investigation, methodology, writing – review and editing. **Yu Fu:** conceptualization, methodology, investigation, funding acquisition, project administration, formal analysis, supervision. **Gillian Vance:** conceptualization, methodology, funding acquisition, supervision, formal analysis, project administration, resources, writing – review and editing.

## Ethics Statement

We received ethical approval from Newcastle University (Number: 24033_3) and approval across four local clinical trusts to recruit nursing and paramedic staff to the study.

## Conflicts of Interest

The authors declare no conflicts of interest.

## Data Availability

Data are not open access to protect participant confidentiality and anonymity. We did not obtain approval from participants or ethics review boards to share data generated by this study openly or beyond the limits of this project.

## Semi‐Structured Interview Questions, Version for New Nurses and Paramedics

**Table:**

Introduction	Tell me a bit about where you have worked during the pandemic?
Overview of experience	Looking back, what are your feelings now about starting work as a < <nurse/paramedic> > at the time when you did, when COVID was new? What are the key moments from the experience you look back on? Did you work in Covid‐intensive areas? What were the biggest challenges? What support did you receive? [Looking back on it all now, would you do it again?]
Wellbeing	How do you feel your wellbeing has been affected? At the time? Now?
	How supported have you felt through this process? [prompt with reference to time off and stress questions]
Career plans	Tell me a bit more about your career plans going forward.
	Have you applied for any further training. Tell me a bit about that decision. When did you make that decision? Do you think your experience during COVID affected your decision?
	Do you think COVID has influenced the < <nurse/paramedic> > you have become/will be? Is that different to the nurse/paramedic you were or anticipated being before covid? What changed, how and why etc? How much do you attribute these changes to the pandemic as opposed to gaining clinical experience? Do you feel these changes are for the better? Whether and how working through COVID changed your views of what it meant to be a < <nurse/paramedic> >?
	Has working through COVID changed your views on working in healthcare/emergency services—what being a < <nurse/paramedic> > is, what medicine can achieve?
Wrap up	Are there any other aspects of your experiences working during Covid that you would like to discuss?
	Do you have any other questions about this study?
